# Research on the comprehensive dessert evaluation method in shale oil reservoirs based on fractal characteristics of conventional logging curves

**DOI:** 10.1038/s41598-025-93224-w

**Published:** 2025-03-18

**Authors:** Li Ju-hua, Zeng Xiao-qin, Lian Cui-hao, Lin Hai, Liu Shiduo, Lei Fengyu, Wan Youyu

**Affiliations:** 1 Hubei Key Laboratory of Oil and Gas Drilling and Production Engineering (Yangtze University), Wuhan, 430100 Hubei Province China; 2 School of Petroleum Engineering, Yangtze University: National Engineering Research Center for Oil & Gas Drilling and Completion Technology, Wuhan City, 430100 Hubei China; 3 Drilling and Production Technology Research Institute, PetroChina Qinghai Oilfield Company, PetroChina, Dunhuang, 736200 China

**Keywords:** Logging curve, Multifractal spectrum, R/S analysis, Fractal dimension, Shale oil, Energy science and technology, Engineering

## Abstract

The traditional logging evaluation of comprehensive sweet spots in shale oil reservoirs has problems such as complex explanatory parameters, incompatible quantitative characterization scales, and low-cost efficiency. A method based on the fractal characteristics of conventional logging curves is proposed to evaluate the comprehensive sweet spots of fractured horizontal wells in shale oil reservoirs. Firstly, the existing evaluation parameters and methods were reviewed, pointing out the limitations of traditional logging evaluation methods. Furthermore, we analyzed 63 fractured sections from three horizontal fractured wells in the Yingxiongling shale oil reservoir of the Qinghai Oilfield, using tracer monitoring data. By applying wavelet transform to reduce noise in high-frequency signals from conventional logging curves, we then used multifractal spectrum analysis and R/S analysis to extract the multifractal spectrum width (∆α) and fractal dimension (D) from four conventional logging attributes: natural gamma logging (GR), acoustic time difference logging (AC), density logging (DEN), and neutron logging (CNL). A multi-attribute comprehensive fractal evaluation index was developed by using the post-fracturing tracer monitoring profile as a constraint and applying the grey relational analysis method. This approach enabled a quantitative classification and evaluation of the key sweet spots in shale oil reservoirs after fracturing. The results show that the comprehensive fractal evaluation index of the high-yield well section after Class I layering is 0.75<∆ α‘<1, 0 < D‘<0.25; 0.35<∆ α‘<0.75, 0.25 < D‘<0.8 in the middle well section of Class II layer; Class III low production well Sect. 0<∆ α‘<0.35, 0.8<∆ α‘<1. Finally, a prediction model for physical property parameters characterized by fractals was introduced using machine learning algorithms, which is 31.9% more accurate than the conventional interpretation physical property parameter prediction model for the comprehensive sweet spot of fracturing. This evaluation method is a concise approach to comprehensively evaluate the sweet spot area based on the extraction of multifractal spectral characteristic parameters from conventional logging data. It is of great significance for characterizing the volume fracturing effect of shale oil and providing technical support for the effective development of shale on a large scale.

## Introduction

China has abundant continental shale oil resources^1–3^. However, due to the development of micro- and nano-scale pores, challenging physical conditions, and significant vertical and horizontal heterogeneity, efficient extraction requires the use of the costly and technically difficult “horizontal wells + volumetric fracturing” approach. As a result, a sweet spot evaluation method for shale oil and gas reservoirs has been developed, which comprehensively considers geological, engineering, and economic factors^3^. Various scholars and oil companies have proposed as many as 52 parameters for evaluating shale oil and gas “sweet spots” based on specific reservoir conditions. This has complicated the evaluation system in practice, leading to significant differences between evaluation results and actual production outcomes^4^. Since China released the industry standard “Shale Oil Geological Evaluation Methods” (GB/T38718-2020) in 2020, key evaluation parameters have been established, including TOC content, thermal evolution maturity, matrix inversion by porosity partitioning, mineral composition and content (lithologic facies), rock mechanical properties (e.g., Young’s modulus and brittleness index), fracture development, residual hydrocarbons (Sl), oil saturation, and formation pressure^5,6^. Quantitative research on these evaluation parameters has led to the creation of a variety of shale oil and gas sweet spot evaluation techniques encompassing seismic, geology, and well logging facets^7–9^. In particular, well logging during drilling serves as an alternative or supplement to indoor core studies and seismic interpretation. It provides continuous geophysical data (e.g., acoustic, electrical, and nuclear properties) with the benefits of lower cost and high vertical resolution. The vertical resolution of this technique ranges from millimeters (e.g., 5 mm for imaging logging) to meters (as seen in conventional logging), improving the linkage between core and seismic data^10,11^.

The shale oil logging technique is mainly based on the qualitative assessment of “seven properties” and “three qualities.” The “seven properties” include lithology, electrical properties, physical properties, hydrocarbon content, hydrocarbon generation capacity, brittleness, and stress-induced anisotropy, while the “three qualities” refer to the quality of the source rock, reservoir, and engineering feasibility^12–14^. However, Chinese continental shale reservoirs are characterized by significant longitudinal variations in lithology, diverse mineralogical components, and the presence of laminae and shale bedding fractures at millimeter to micron scales. These factors make logging identification and quantitative analysis more challenging^14,15^. Furthermore, conventional anisotropic interpretive models are unable to accurately characterize the true longitudinal anisotropy. Despite advancements such as two-dimensional nuclear magnetic resonance (NMR) for mobile full-diameter cores, four-dimensional digital core simulation and analysis techniques, and high-precision elemental logging of rock mineralogical fractions^16–18^, these logging methods still ultimately rely on high-precision petrophysical experimental results^19,20^. The thin interbedding of shale reservoirs, along with their high variability in both vertical and horizontal distribution, makes it challenging to accurately capture their physical characteristics using discrete centimeter-scale core samples. This leads to problems in prediction accuracy and the continuity of interpreted regions when using interpretation templates that depend on the results of indoor experiments to calibrate logging curves.

Fractal theory, as a mathematical method with unique advantages in characterizing complex irregularities, has been increasingly used in the field of geosciences. Studies have shown that the microstructure of shale reservoirs satisfies fractal features^21–23^ utilized multiple fractal parameters from T2 spectra in NMR experiments to highlight differences in rock pore structure and to classify the types of pore structures^24^. Classified the pore structure of carbonate rocks by analyzing the fractal dimension of logging curves. They used the weighted box dimension of porosity curves and the weighted R/S dimension rendezvous plot of resistivity curves to establish the criteria for classifying pore structures^25^. Established a test-well interpretation model for multi-stage fractured horizontal wells in fractured shale gas reservoirs and analyzed the influence of parameters such as fractal index and fractal dimension on pressure dynamics^26^. Explored the fractal characteristics and thermal evolution laws of shale pores using the FHH fractal model. Their work used fractal theory to make significant progress in the quantitative characterization of the physical properties of shale reservoirs.

Given the limitations of conventional well logging evaluation methods—such as regional geological influence, prediction accuracy, and parameter complexity—this study proposes a new comprehensive method for evaluating sweet spot sections in fractured horizontal wells within shale reservoirs. Building on the qualitative insights provided by conventional well logging curves, this method integrates multi-attribute well logging curves with meter-level accuracy and production profile data from post-pressure tracer concentration monitoring. The goal is to more effectively extract the physical parameters of shale reservoirs. This study established a comprehensive fractal evaluation method by extracting multiple fractal spectra and R/S fractal dimensions from conventional well logging curves, which were then used to classify and evaluate the production level of the sweet spot section in fractured horizontal wells in shale reservoirs. In this study, we use the CP2 and CP4 wells, located in the sweet spot of the 14–15 box in the Hero Ridge shale reservoir in the Qinghai Oilfield, as examples. The multiple fractal spectra are firstly applied to extract four logging attribute curves of the target wells, such as natural gamma logging (GR), electrical resistivity logging (AC), density logging (DEN), and neutron logging (CNL), and then analyze fractal characterizations of qualitative analysis of reservoir heterogeneity, pore and fluid distribution on the basis of the fractal dimension and the oil-phase dimension of the wells. Based on the qualitative analysis of the fractal characterization of reservoir inhomogeneity, pore and fluid distribution, and combined with the grading results of oil-phase tracer monitoring in oil wells, the gray correlation method was adopted to determine the ∆α’ and D’ values of the combined weights of fractal spectral width ∆α and fractal dimension D of the multi-attribute logging curves. By effectively integrating the geological attributes revealed by well logging curves, a comprehensive evaluation index system for shale oil classification was finally constructed. The system quantitatively characterizes the classification and evaluation criteria based on well logging curve fractals. This evaluation method addresses the limitations of traditional approaches and enables capacity grading of shale oil formations at comprehensive sweet spots after pressure testing. This method provides simple and necessary technical support for the selection of sweet spot zones in fractured shale oil wells, and has important practical application value.

## Comprehensive sweet spots routine evaluation of Heroes’ Ridge shale reservoirs

### Conventional evaluation criteria for integrated sweet spots in the Heroes’ Ridge Shale

The shale oil reservoir in the Hero Ridge E32IV-VI oil group of the Qinghai Oilfield has been vertically divided into 23 distinct layers, each characterized by unique mixed sedimentation^27–29^. Based on sedimentary structure, laminae thickness, and mineral components, these layers are classified into six lithological types: laminated limestone, laminated dolomite limestone, layered dolomite, layered dolomite limestone, layered clay shale, and layered mudstone.In evaluating and selecting vertical sweet spot sections and planar sweet spot areas for the Hero Ridge shale oil reservoir, the following key factors are considered: source rock quality, reservoir quality, engineering quality, and flow quality. These factors are essential for optimizing exploration and development strategies.

Conventional logging can provide a qualitative and comprehensive evaluation of shale reservoir pore structure, natural fracture complexity, fluid distribution, and oil content conditions. The total organic carbon content (TOC) and hydrocarbon production potential (S1) of rock pyrolysis, which are indicators for evaluating the organic matter abundance of hydrocarbon source rocks, are not obtained directly from logging curves, but are obtained from laboratory core analysis, which indirectly establishes the correlation with certain logging curves. For example, clay is correlated with organic matter through natural gamma logging (GR); resistivity logging (AC) reflects the porosity and fluid type of the formation and infers the presence of organic matter; density logging (DEN) provides the porosity and mineralogical composition of the formation’s rocks, indirectly correlating with the organic matter content; neutron logging (CNL) reflects the formation’s hydrogen content, which can indirectly indicate the type of pore fluids, including the presence of organic matter; elemental logging can indirectly infer organic matter content by measuring specific elements (e.g., carbon, sulfur) in the rock; and nuclear magnetic resonance (NMR) logging can provide information about pore fluids and pore structure of the rock, which can help in assessing the organic matter content of the rock.

Although the above logging curves can provide indirect information about formation properties, they usually need to be interpreted comprehensively in conjunction with core analysis and other geological data. Based on a comprehensive analysis of logging, oil test, and production test data, the key parameters for oil enrichment in the Heroes’ Ridge Shale were identified as TOC, S1, porosity, saturation, and compressibility index. Subsequently, three types of sweet spot classification and evaluation criteria were established, as detailed in Table 1.

**Table 1:** Sweet spot characteristic evaluation standards of Yingxiongling shale oil.

Criteria for classification	Category I	Category II	Category III
Main lithologies	Laminated gray dolomite, grainy cloudy limestone	Laminated gray dolomite, grainy cloudy limestone	Striated gray dolomite, striated clayey shale
TOC	> 1.0%	0.6–1.0%	0.4–0.6
S1	> 3 mg/g	1–3mg/g	< 1 mg/g
Effective porosity %	≥ 6	4–6	< 4
100ms cutoff % porosity	≥ 2	0.5–2	–
NMR permeability/mD	> 0.1	> 0.02	> 0.01
Clay mineral content/%	< 20	< 35	< 40
brittleness	Good	Good	Medium/Poor
Development of the lamellar suture	well	better	poor
source storage factor	1.2–1.5	1-1.2	< 1

In the process of evaluating the comprehensive sweet spot of shale reservoirs, conventional logging evaluation techniques encounter many problems. For example, the evaluation parameters are complicated and numerous, and the calculation process is intricate, which itself increases the complexity of the work. Moreover, obtaining these evaluation parameters often requires reliance on expensive indoor experiments and field tests. Additionally, the interpretation templates of conventional logging curves are mostly based on discontinuous centimeter-scale core samples, which limits their ability to comprehensively characterize reservoir properties. By combining production profile data with tracer concentration monitoring from post-pressure wells, it becomes clear that areas categorized as Class I composite sweet spots are not necessarily the fractured sections that contribute the most to production. These observations highlight the shortcomings of conventional logging evaluation methods in quantitative categorization, which in turn affects their utility and accuracy in guiding effective volumetric fracturing.

###  Comprehensive sweet spots post-pressure effect analysis of Heroes’ Ridge Shale reservoirs

The oil-phase tracer monitoring horizontal well production profiles were used to evaluate and analyze the post-compression effect of the fractured horizontal wells CP2 and CP4 in the middle-sweet spot zone of the 14–15 box of the Heroes’ Ridge shale reservoir. The oil production contribution rate was determined based on the percentage of oil and water phase tracer content in the CP2 and CP4 wells. The dimensionless productivity rate of each fractured horizontal well was calculated based on the average oil production per section. A dimensionless productivity rate greater than 1.2 times the datum value was classified as high yield (Class I)^30^. A rate less than 0.6 times the datum value was classified as low yield (Class III). Rates between 0.6 and 1.2 times the datum value were classified as medium yield (Class II). The data processing results are shown in the dimensionless productivity capacity grading table for shale oil wells in Table 2.

**Table 2:** Shale oil well dimensionless production grading scale.

Pound sign	Fracturing section	Water phase	Oil phase	Oil phase contribution	Dimensionless productivity	Pound sign	Fracturing section	Water phase	Oil phase	Oil phase contribution	Dimensionless productivity
CP 2	1	3.8	4.9	0.56	1.14	CP 4	2	4	3.2	0.44	0.86
	2	3	5.9	0.66	1.34		3	1.7	6.2	0.78	1.52
	3	2.3	4.9	0.68	1.37		4	8.8	8.2	0.48	0.93
	4	3.6	4.3	0.54	1.1		5	5.3	4.7	0.47	0.91
	5	3	5	0.63	1.27		6	4.3	3.5	0.45	0.88
	6	3.5	5.3	0.6	1.21		7	2.5	4.3	0.63	1.23
	7	4	3.5	0.47	0.95		8	6.2	6.5	0.51	0.99
	8	6.2	1	0.14	0.28		9	7.5	3.5	0.32	0.62
	9	5	3.6	0.42	0.84		10	8.7	3	0.26	0.51
	10	4.1	5.5	0.57	1.16		11	4.1	6.8	0.62	1.21
	11	3.7	4.2	0.53	1.07		12	3.5	4	0.53	1.03
	12	3.2	6.6	0.67	1.36		13	3	9	0.75	1.46
	13	7.3	6	0.45	0.91		14	7.3	6.2	0.46	0.89
	14	6.8	2.2	0.24	0.49		15	6	4.1	0.41	0.8
	15	6.1	4.2	0.41	0.82		16	9.5	5.6	0.37	0.72
	16	8.5	13	0.6	1.22		17	4.2	5.8	0.58	1.13
	17	4.8	12.7	0.73	1.47		18	2.9	5.2	0.64	1.25
	18	11.3	3.4	0.23	0.46		19	2.1	4.2	0.67	1.3
	19	9.1	3.4	0.27	0.55		20	1	2.7	0.73	1.42

1 $$\:\text{q}=\frac{{\text{q}}_{\text{o}}}{\stackrel{-}{{\text{q}}_{\text{o}}}}$$

q is the dimensionless productivity, decimal; q_o_ is the segmental oil production, t/d; and $\:{\text{q}}_{\text{o}}$is the average segmental oil production, t/d.

**Fig. 1:**
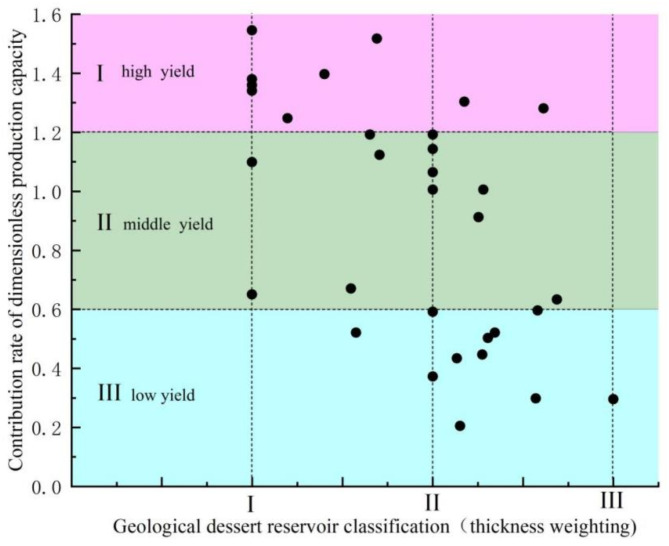
Plate of the relationship between the integrated sweet spot section and the corresponding dimensionless capacity contribution of the fractured horizontal wells in the middle sweet spot area of Hero Ridge.

Additionally, by incorporating the effective thickness weighting of each fracturing section and applying the three types of sweet spot classification criteria in Table 1, we plotted the relationship between the weighted grading of geological sweet spot reservoirs and the post-fracturing production profiles of wells in the 14–15 box area, as shown in Plate 1. As shown in Fig. 1, the dimensionless production of fracturing sections in Class I sweet spot reservoirs (weighted thickness), evaluated according to the parameters in Table 1, is at high and medium levels. In contrast, the dimensionless production for Class II sweet spot reservoirs (weighted thickness) is more variable, with production spread across high, medium, and low ranges. The dimensionless production of Class III sweet spot reservoirs (weighted thickness) is generally low, with only a few instances at high levels. The dimensionless yield shows a scattered distribution, being distributed in high, medium, and low intervals. The dimensionless yield of the fractured section of Class III sweet spot reservoirs (weighted thickness) is basically low, with only a few instances in the high yield category.

In general, the geological sweet spots established with the help of the parameters in Table 1 have a certain correspondence with the graded contribution of oil production from fractured wells. According to specific analysis, the dimensionless production of the fracturing section corresponding to the Type I sweet spot is usually located in the high-yield or medium-yield area and has a relatively stable output performance. However, for the fracturing section in the transition zone between Type I and Type II, and Type II and Type III sweet spot reservoirs (weighted thickness), the dimensionless production distribution is relatively scattered and uncertain. The dimensionless production appears in high-yield, medium-yield, and low-yield areas and lacks clear regularity.However, due to limitations in the accuracy of conventional logging interpretation methods, the contribution of oil production from fractured wells is very low in some of the geological Class II sweet spots. Conventional sweet spot evaluation methods can no longer meet the exploration and development needs of unconventional reservoirs that require large investments. The author adopts the multiple fractal spectrum extraction method to continuously characterize multiple attributes from logging curves and combines them with the constraints of production profile test data with meter-scale accuracy to propose a comprehensive multiple fractal evaluation method for shale reservoirs with complex non-homogeneous distribution. This method eliminates the tedious process of calculating multiple physical property parameters during logging interpretation, reduces the cost of indoor high-precision core experiments, and organically combines engineering sweet spots and geological sweet spots with reservoir production capacity. This is of significant value in engineering applications.

##  Establishment of fractal characterization methods for logging curves

### Preparation for standardization of logging data

In order to eliminate the influence of singular sample data, it is necessary to standardize the logging data.

2 $$\:\begin{array}{c}{\text{X}}_{\text{i}}=\frac{{\text{x}}_{\text{i}}-{\text{x}}_{\text{m}\text{i}\text{n}}}{{\text{x}}_{\text{m}\text{a}\text{x}}-{\text{x}}_{\text{m}\text{i}\text{n}}}\end{array}$$

In the above equation, $\:{\text{X}}_{\text{i}}$ is the data value after normalization, xi is the original logging data value, and x_max_ and x_min_ are the maximum and minimum values in the original logging data, respectively.

### Pickup of multiple fractal spectra of logging curves

The pore structure and fluid distribution of the reservoir are not homogeneous, and there exists only an approximate self-similarity in the statistical sense^31,32^. A multifractal method can be used to establish the fractal dimensions of a number of different fractal subsets for a comprehensive identification and description of the pore structure and fluid distribution at different scales.

First, the actual logging values are mixed with the noise spectrum, necessitating noise elimination through wavelet analysis. A wavelet function with volatility and attenuation characteristics is selected for wavelet decomposition of the logging curve. The volatility characteristic requires that the higher-order moments of the wavelet function are zero, i.e.:

3 $$\int_{{ - \infty }}^{{+\infty }} {{x^k}\psi (x)dx=0,{\text{ }}(k=0,…,N - 1} )$$

At this point, it is referred to as an Nth order vanishing moment wavelet, and its Fourier spectral function is Nth differentiable. Attenuation requires that the domain of definition of the wavelet function is compactly supported to ensure its rapid decay properties.

Signals of finite energy$f(x) \in {L^2}(R)$of the wavelet transform as:

4 $${W_f}(a,b)=\langle f,{\psi _{a,b}}\rangle =\int_{{ - \infty }}^{{+\infty }} {f(x)\psi _{{a,b}}^{*}(x)dx=} \int_{{ - \infty }}^{{+\infty }} {f(x)\frac{1}{{\sqrt a }}} {\psi ^*}(\frac{{x - b}}{a})dx$$

where a is the scale parameter.$a \in R$,and$a \ne 0$,b is the translation parameter,$b \in R$. a, b constitutes the phase space of scale-localized positions.

For multifractals, there are some modulus maxima of their wavelet transforms. From Eq. (4):

5 $$\left| {{W_f}} \right.\left. {(a,b)} \right|\sim {a^{\alpha +\frac{1}{2}}}$$

$\:{\upalpha\:}$ is the singularity index, from Eq. (5), the above equation holds when the wavelet function satisfies the order vanishing moment. The larger the singularity of the analyzed signal is the higher the order of the required vanishing moment, the smoother the wavelet is Daubechies wavelet as a tightly supported orthogonal wavelet has enough high order vanishing moments and symmetry, which can effectively avoid the phase distortion, and db3 is selected as the orthogonal wavelet basis and then reconstructs the signal by the low-frequency coefficients obtained through wavelet decomposition and high frequency coefficient reconstruction signal obtained by thresholding quantization processing to achieve the Wavelet denoising goal^33^.

Multifractal singular spectrum is obtained using wavelet modulus maxima transform. Firstly, the waveform data are polarized to obtain the corresponding extreme value points, and db3 is used as the orthogonal wavelet basis to measure all the singularity indices. The partition function at each scale is then calculated and linearly regressed.

6 $${\log _2}Z(q,a) \approx \tau (q){\log _2}a+C(q)$$

7 $$\tau (q)=\hbox{min} \left( {q\left( {\alpha +\frac{1}{2} - f(\alpha )} \right)} \right)$$

Find the spectrum of multifractal singularities:

8 $$f(\alpha )=\hbox{min} \left( {q\left( {\alpha +\frac{1}{2} - \tau (q)} \right)} \right)$$

9 $$\Delta \alpha ={\alpha _{\hbox{max} }} - {\alpha _{\hbox{min} }}$$

10 $$\Delta f(\alpha )=f({\alpha _{\hbox{max} }}) - f({\alpha _{\hbox{min} }})$$

The four main fractal extraction properties that characterize the multifractal parameters are the spectral width ∆α, the multifractal spectrum f(α)max, the maximum and minimum probability subset fractal dimension difference ∆ f (α), and the symmetry parameter |B|.

### Determination of the dimensionality of the R/S variable scale fractal

R/S analysis^34^, or variable scale analysis, is suitable for analyzing data with continuous volatility. In the field of logging, the logging values at different depths are used as variables to form a sequence that varies with logging depth. The heterogeneity of logging data can be evaluated by the R/S fractal dimension, and the larger the fractal dimension, the stronger the heterogeneity^35^.

To perform R/S analysis, it is first necessary to find the mean value of the logging data, and then calculate the accumulated deviation of the sampling points in each stratum section.

11 $$Z(u,N)=\sum\limits_{{n=1}}^{u} {\left\{ {x(n) - \overline {{x(n)}} } \right\}} {\text{ }}(1 \leqslant u \leqslant N,n=1,2,…,N)$$

The extreme deviation is then derived from the accumulated deviation, which reflects the degree of variation in the logging curve.

12 $$R(N)=\mathop {\hbox{max} }\limits_{{0<u<N}} Z(u,N) - \mathop {\hbox{min} }\limits_{{0<u<N}} Z(u,N)$$

Finally, the standard deviation is then taken, which reflects the degree of deviation of each sampling point from the mean and characterizes the volatility of the overall logging curve.

13 $$S(N)=\sqrt {\frac{1}{N}{{\sum\limits_{{u=1}}^{N} {\left[ {x(u) - \overline {{x(n)}} } \right]} }^2}}$$

Then divide and get the dimensionless ratio R/S, and then perform linear regression on log(N) ~ log(R/S) in double logarithmic coordinate system. When R/S is approximately linear with N, it indicates that the logging curve has fractal characteristics and the relationship exists, so the fractal dimension D can be obtained.

### Establishment of comprehensive fractal evaluation indicators

Taking CP 2 and CP 4 wells as examples, the fractal-related information of AC, CNL, DEN, and GR logging attribute curves of each fractured well section is picked up respectively, and the fractal attributes of the four logging curves are calculated to compare with the dimensionless production, and the statistical results are shown in Table 2. Since the four conventional logging curves respectively reflect reservoir attributes to get numerous fractal parameters, it is necessary to establish a comprehensive fractal characterization considering the weighting level. Accordingly, the gray correlation analysis was used in this study, with the dimensionless production of CP 2 and CP 4 wells as the parent sequence xi0(i = 1,2,3…n), and the fractal spectral width ∆α value and fractal dimension D value of each logging curve as the subsequence x_ij_ (i = 1,2,3…n; j = 1,2,3 …m) jointly to determine the gray correlation coefficient.

14 $$\Delta X=\left| {{x_{ij}}} \right.\left. { - {x_{i0}}} \right|$$

15 $$\Delta {X_{\hbox{max} }}=\hbox{max} \left| {{x_{ij}}} \right. - \left. {{x_{i0}}} \right|$$

16 $$\Delta {X_{\hbox{min} }}=\hbox{min} \left| {{x_{ij}}} \right.\left. { - {x_{i0}}} \right|$$

17 $${R_{co}}=\frac{{\Delta {X_{\hbox{min} }}+\delta \Delta {X_{\hbox{max} }}}}{{\Delta X+\delta \Delta {X_{\hbox{max} }}}}$$

where: is the absolute difference between the factors of the subsequence and the parent sequence, and are the maximum and minimum of the absolute difference, respectively;$\:{\text{R}}_{\text{c}\text{o}}$is the gray correlation coefficient,$\:{\updelta\:}$ is the discriminant scale, used to reduce the impact of the absolute difference is too large; the value of $\:{\updelta\:}\:\text{i}\text{s}\:\text{b}\text{e}\text{t}\text{w}\text{e}\text{e}\text{n}\:$[0.1,1], this time to take 0.5, so that the evaluation of gray correlation coefficient is more significant.

Firstly, the grey incidence coefficient is calculated:

18 $${R_{j,0}}=\frac{1}{n}\sum\limits_{{i=1}}^{n} {{R_{co}}}$$

The closer the gray correlation is to 1, the more closely the subsequence is linked to the parent sequence. The degree of influence of different sub-sequences on the parent sequence can be determined based on the ordering of the gray correlation.

The weight coefficients are then calculated by normalizing the gray correlation:

19 $${\omega _j}=\frac{{{R_{j,0}}}}{{\sum\nolimits_{{j=1}}^{m} {{R_{j,0}}} }}$$

The grey correlation coefficients were further determined and normalized according to the grey correlation coefficients between the partial factors of the sub-sequence and the partial factors of the parent sequence to obtain the weighting coefficients about ∆α and D. The statistical results are shown in Table 3 and 4.

**Table 3:** Fractal-related parameters and dimensionless production statistics for fractured sections of CP2 and CP4.

CP 2 well							CP 4 well					
Fracturing section	Causality	AC	CNL	DEN	GR	Dimensionless productivity	Fracturing section	Causality	AC	CNL	DEN	Dimensionless productivity
19	∆α	0.04	0.198	0.009	0.215	0.55	20	∆α	0.016	0.093	0.004	1.42
	∆f(α)	0.316	−0.202	−0.07	−0.249			∆f(α)	0.317	−0.076	−0.021	
	D	1.505	1.314	1.435	1.358			D	1.552	1.417	1.475	
	B	−0.01	0.064	0.001	0.037			B	−0.006	0.009	0	
18	∆α	0.023	0.031	0.001	0.123	0.46	19	∆α	0.01	0.119	0.006	1.3
	∆f(α)	0.037	−0.308	0.013	0.284			∆f(α)	−0.085	−0.064	−0.04	
	D	1.533	1.53	1.539	1.473			D	1.465	1.402	1.489	
	B	−0.001	0.007	−0.001	−0.039			B	0.001	0.002	0.001	
17	∆α	0.029	0.051	0.002	0.102	1.47	18	∆α	0.004	0.107	0.007	1.25
	∆f(α)	0.178	0.131	−0.033	−0.043			∆f(α)	0.042	−0.042	0.001	
	D	1.536	1.52	1.499	1.49			D	1.485	1.486	1.411	
	B	−0.005	−0.009	0	−0.012			B	0	0.009	0.001	
	–							….				
2	∆α	0.016	0.036	0.003	0.14	1.34	3	∆α	0.047	0.049	0.002	1.52
	∆f(α)	−0.03	−0.492	0.041	−0.584			∆f(α)	−0.397	−0.303	0.005	
	D	1.459	1.53	1.524	1.438			D	1.415	1.533	1.473	
	B	0.002	0.014	0.001	0.076			B	0.013	0.009	0	
1	∆α	0.03	0.184	0.006	0.171	1.14	2	∆α	0.044	0.11	0.003	0.86
	∆f(α)	−0.039	−0.03	−0.049	0.022			∆f(α)	−0.009	0.04	−0.013	
	D	1.498	1.502	1.472	1.568			D	1.408	1.407	1.454	
	B	0	−0.02	0	−0.035			B	−0.002	−0.006	−0.001	

**Table 4:** Grey relational degree of multifractal spectrum width ∆α and fractal dimension D.

causality	AC	CNL	DEN	GR
Spectral width ∆α	0.2472	0.2442	0.2490	0.2596
Dimension D	0.2501	0.2618	0.2457	0.2424

## Comprehensive fractal evaluation method validation and application

###  Establishment of comprehensive fractal metrics for volumetric fracturing of shale oil

According to the method of Sect. 2, the flow chart of this study is as follows :


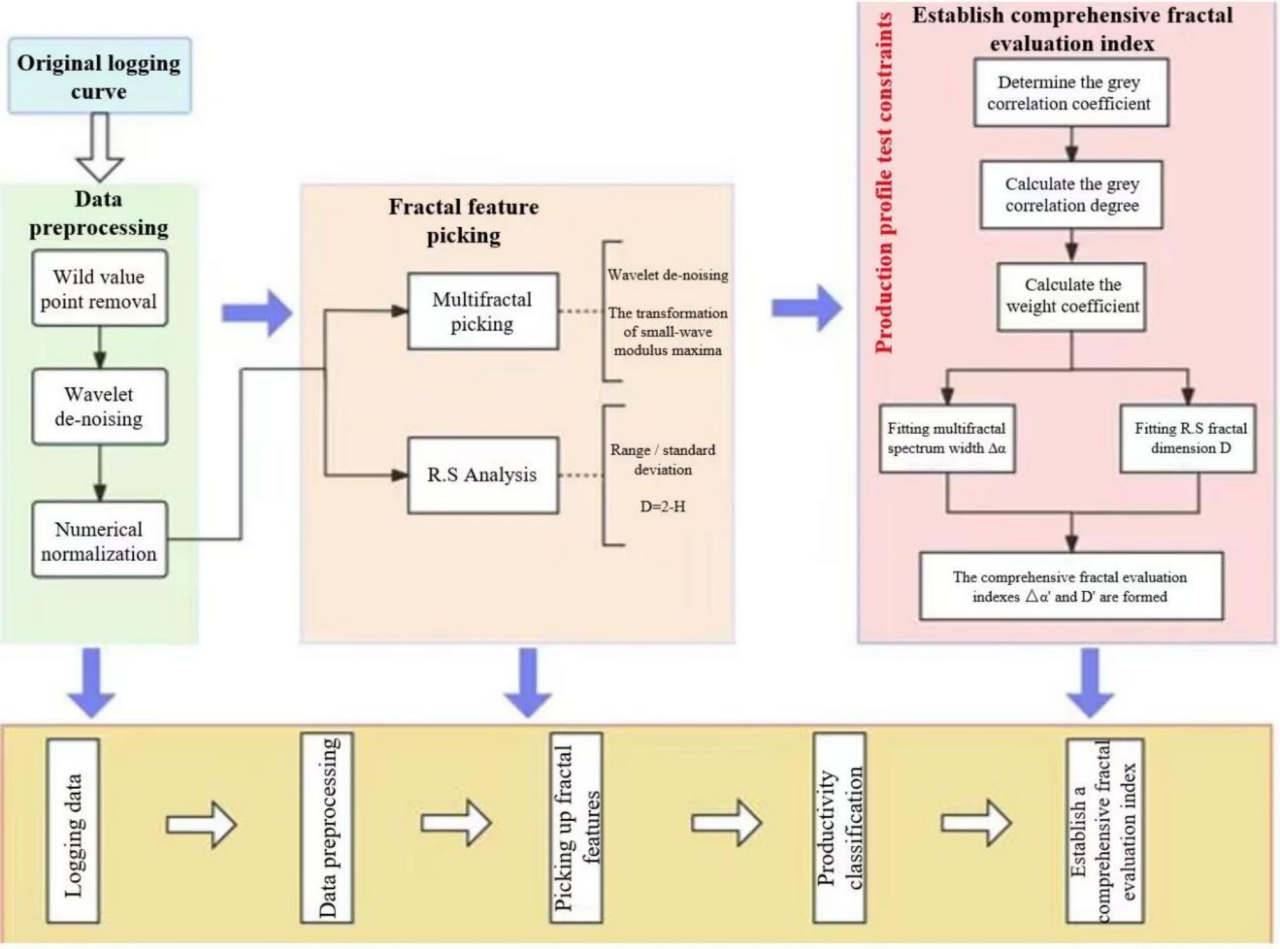


**Fig. 2:**
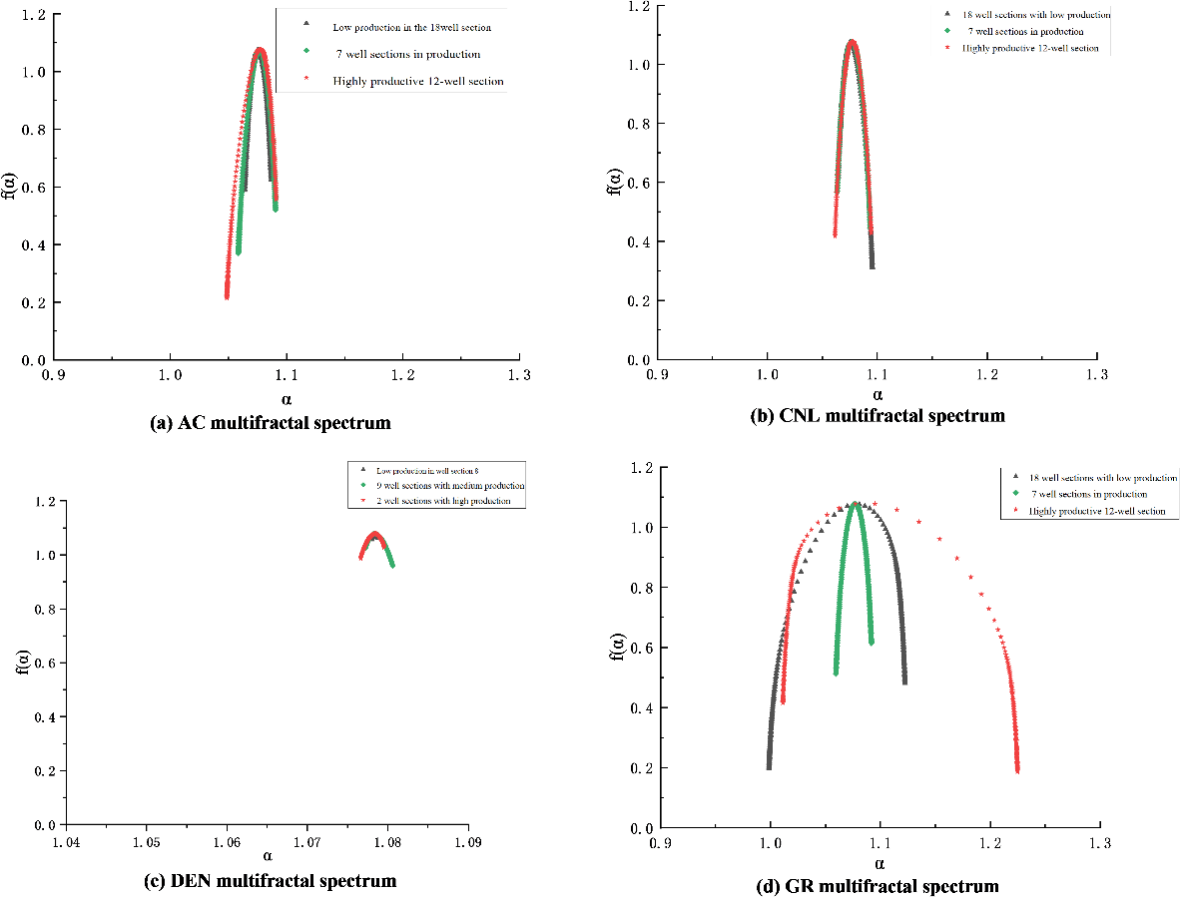
Four-attribute multifractal spectrum of logging in different fractured sections of CP 2 wells.

Taking CP2 well as an example, according to the statistics of fractal attributes and dimensionless yield classification in Table 2, the corresponding four-attribute multiple fractal spectra of the conventional logging curves were drawn schematically by selecting the fractured sections with high, medium, and low levels of yield classification, respectively, as shown in Fig. 2.

Combining the fractal characteristics of the fractured section capacity grading of CP 2 wells reflected in Table 2; Fig. 2, there are mainly the following insights:

1. The multifractal spectrum width ∆α of GR logging curves reflecting shale clay content and organic matter properties is the largest, and the multifractal spectrum width ∆α of DEN logging curves reflecting shale pore structure ( DEN, CNL, AC ) is the smallest, indicating that there is no significant difference in the development degree of nanopore structure. The multifractal spectrum f(α) max of the four logging attributes is not much different, indicating that the peak values of the four attributes are not significantly different.
2. The fractal dimension D values of the attribute parameters extracted from the four logging channels by the R/S technique are more consistent in the fracturing sections corresponding to high, medium, and low production capacities. Specifically, the fractal dimension D of the high-production section is lower than that of the low-production section. The fractal dimension D values for each attribute are as follows: AC corresponds to 1.535 for the high-production section and 1.585 for the low-production section; CNL corresponds to 1.409 for the high-production section and 1.603 for the low-production section; DEN corresponds to 1.524 for the high-production section and 1.594 for the low-production section; GR corresponds to 1.524 for the high-production section and 1.594 for the low-production section. The overall fractal dimension D value is 1.438 for the high-production section and 1.566 for the low-production section, indicating that the pore structure of the reservoir in the high-production section is more uniform than that in the low-production section. This suggests that the reservoir in the high-production section is more homogeneous, with higher permeability and higher flowable oil saturation in the nanopore space.

In summary, selecting the multiple fractal spectral width ∆α and fractal dimension D values among the fractal attributes that are more sensitive to reflecting the production capacity level can qualitatively evaluate the fracturing production capacity grading of shale oil wells. In order to comprehensively consider the weights of the signals of each logging attribute, gray correlation analysis is used to fit the ∆α’ value and D’ value according to the weight coefficients. Taking CP 2 and CP 4 wells as an example, the comprehensive evaluation indexes of the training set (60% of the data points are selected) and the testing set (40% of the data points) are calculated respectively by the above method to plot the comprehensive scores. Comprehensive fractal evaluation method is an efficient and low-cost shale oil well capacity evaluation technique. By analyzing the fractal features of logging data and combining with gray correlation analysis, it can quickly and accurately evaluate the production capacity of shale oil wells in a hierarchical manner. The method shows significant cost advantages and efficiency improvement potential in practical applications, and is particularly suitable for the development and management of unconventional oil and gas reservoirs.

**Fig. 3:**
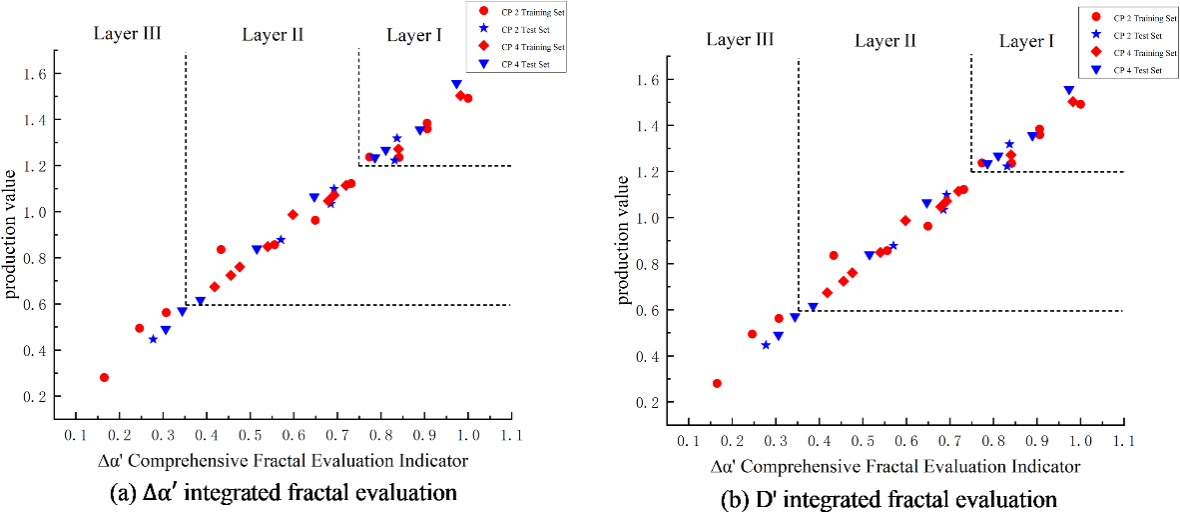
Graph of integrated fractal evaluation index for weighted fractal parameters.

A graphical version of the shape indicator is shown in Fig. 3. In the graphical version of Fig. 3 Comprehensive Fractal Evaluation, red and blue colors are used to distinguish the evaluation metrics for the training and test sets, respectively: the training set metrics are marked with red dots, while the test set metrics are represented by blue dots, allowing for the intuitive observation of a close relationship between the fitted and predicted comprehensive fractal evaluation metrics and the dimensionless yield.

The level of fracturing section capacity at different geologic sweet spots is meticulously divided into three categories, each with a unique range of evaluation metrics:

1. Class I fractured high-yield section: the comprehensive fractal evaluation index is defined between 0.75 and 1, while the value of fractal dimension D’ is between 0 and 0.25. The index in this interval reflects that the reservoir in the high-yield section has a high degree of pore homogeneity and good permeability
2. Class II fractured medium-producing section: the comprehensive fractal evaluation index ranges from 0.35 to 0.75, while the value of fractal dimension D’ is between 0.25 and 0.8. The indicators in this interval reveal that the pore structure and permeability of the reservoir are in a transitional state between high- and low-production well sections.
3. Class III fractured low-productivity section: the comprehensive fractal evaluation indexes range from 0 to 0.35, while the value of fractal dimension D’ is higher than the range of 0.8 to 1. These indicators suggest that the reservoir pore structure and permeability are relatively poor in the low-producing well sections.

The overall trend shows that the comprehensive fractal evaluation index ∆α’ of high-producing wells is close to 1, and this index shows a decreasing trend with decreasing production. Comparatively, the integrated fractal evaluation index D’ of R/S subdimension for low-production wells is also close to 1, and this indicator shows a decreasing trend as the yield rises. The conclusions of the training set and the test set are highly consistent, which not only verifies the validity of the method, but also demonstrates its reliability in practical applications.

### Method validation

**Fig. 4:**
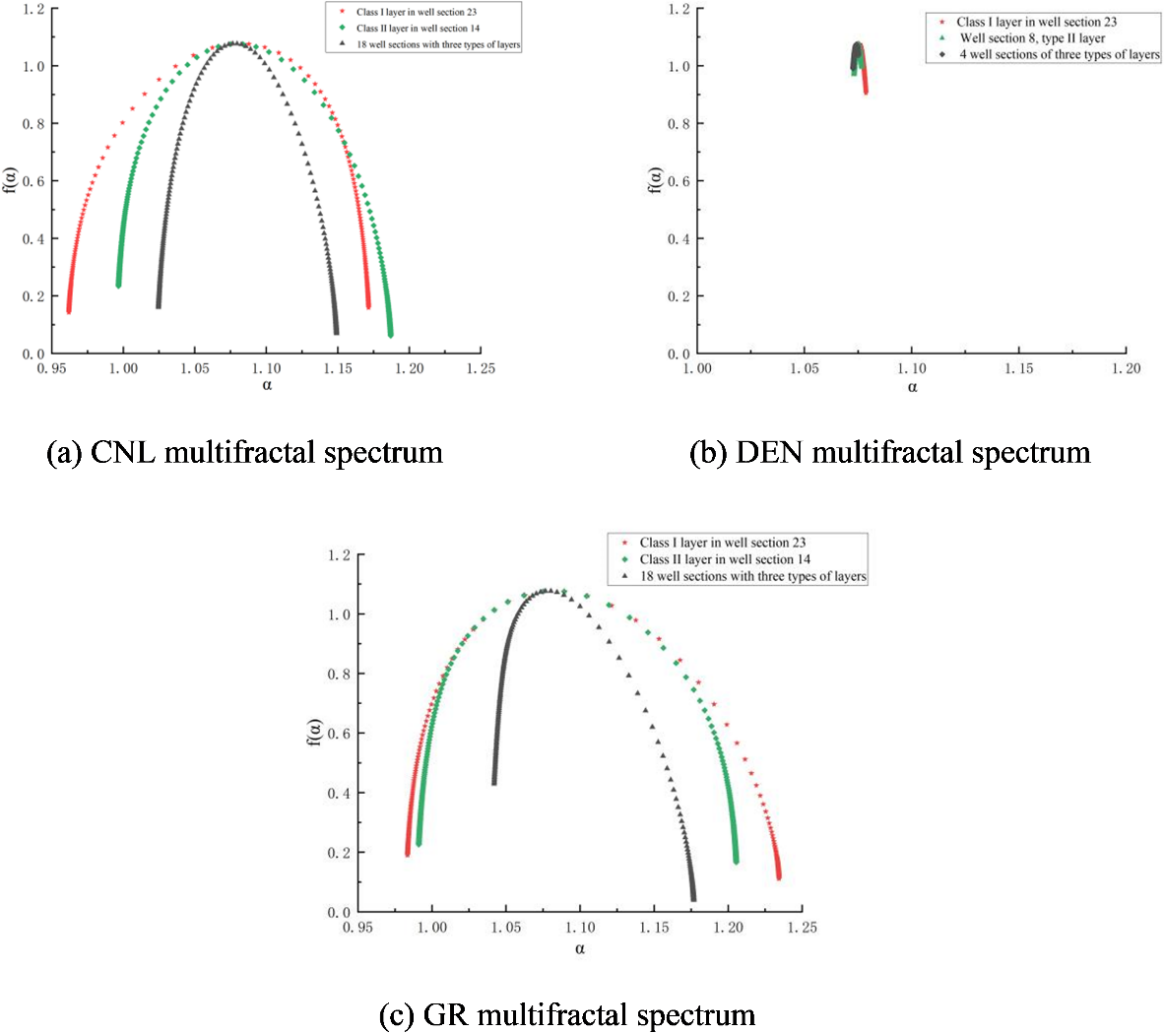
Multifractal spectrum of different well sections of YYH14-2 fractured horizontal well.

This fractal evaluation method was applied to the tracer-monitored fluid-producing profile of the Yangpai 14 − 2 well, located in the sweet spot two platform of the Hero Ridge shale reservoir. First, the multiple fractal spectrum of the conventional logging curve for the multi-segment fractured section of this well is calculated^10^. Although the AC logging curve is missing from this well test, its pore and fluid information is captured by the other three attribute logging curves. Therefore, the absence of the AC logging curve does not significantly impact the prediction results. Calculating the fractal characteristics of the other three logging attributes, as shown in Fig. 4, shows that the second platform’s reservoir has the following fractal characteristics:

1. The multiple fractal spectral width ∆α of attribute GR is the largest, and that of attribute DEN is the smallest, indicating that the reservoir has a higher degree of fracture development and contains fluid, which reflects more sensitively to the degree of irregularity in the fractal structure of the extracted attribute signals.
2. The symmetry parameter B < 0 of the multifractal spectrum of attribute CNL shows that the right skewed spectrum has a larger fractal dimension index, stronger fractal intensity, and denser fracture development.
3. The symmetry parameter B > 0 of the multifractal spectrum of attribute GR shows that the dimension index of the left skewed spectrum is lower, and the fractal intensity is weaker, which indicates that the non-homogeneity of the reservoir is weaker, and it is empirically proved that the multifractal characteristics basically conform to the multifractal law of volumetric fracturing summarized in the previous section.

**Fig. 5:**
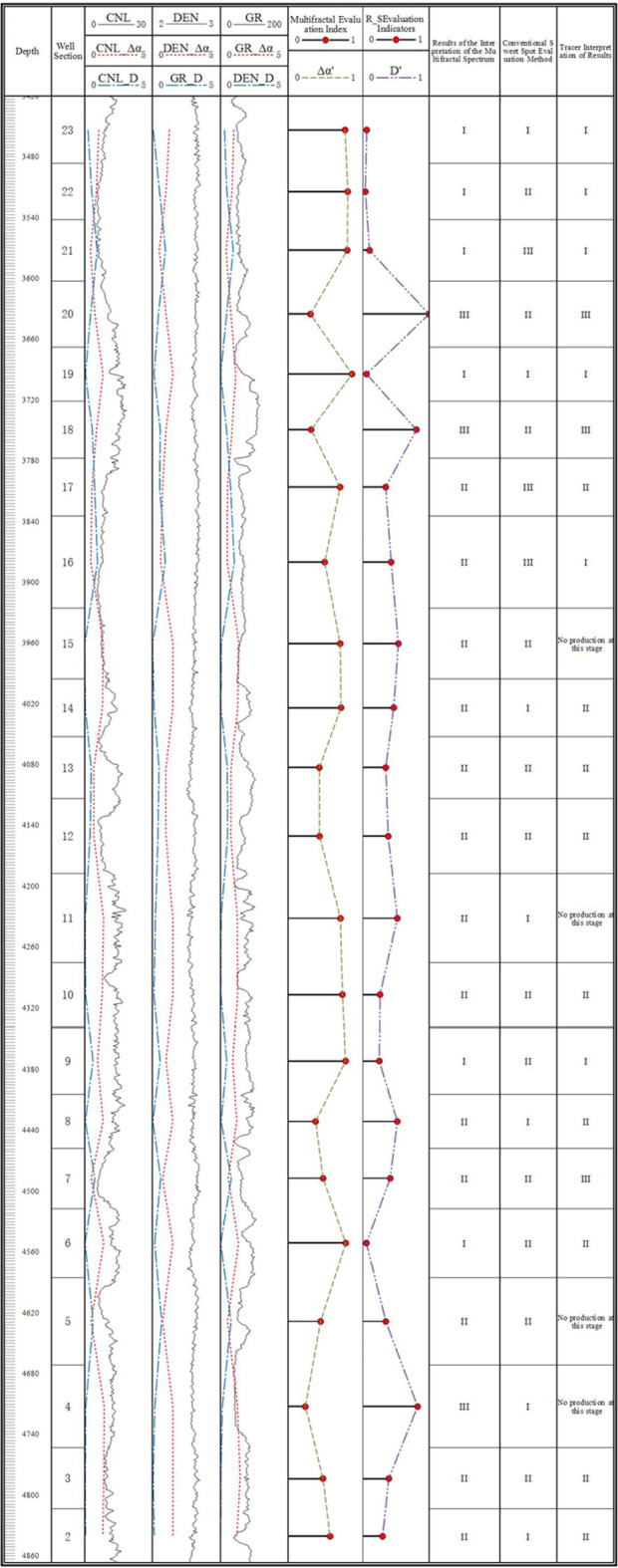
Graph of well log and comprehensive fractal evaluation index relationship.

Comprehensive fractal evaluation indicator plate, drawing the fractal characterization and prediction of well logging curves of Yangpai 14 − 2 well comprehensive sweet spots Fig. 5 shows that the comprehensive fractal evaluation indicator ∆α’ of the high-yielding wells is close to 1 and shows a decreasing trend from high-yielding to low-producing; the comprehensive fractal evaluation indicator D’ of the R/S fractional dimension of the low-producing wells is close to 1 and shows a decreasing trend from low-producing to high-yielding. D’ is close to 1, and shows a decreasing trend from low to high production. When compared to conventional geological sweet spot evaluations and production profile grading, this method shows a high overall match with the fractured section. Out of the fractured sections, six segments of high-yielding Class I layers were accurately predicted with an accuracy of 83.3% using this method. In contrast, the geological sweet spot criterion only achieved a 16.7% accuracy for the prediction of one high-yielding layer segment. Nine segments of the medium-producing Class II layers were fractured, with this method reaching a prediction accuracy of 88.9%. In contrast, using the geological sweet spot criterion, the prediction accuracy for four segments of the medium-producing layers was only 44.4%. Fracturing and tracer data for the low-producing layers reveal seven non-producing segments. This method achieved a prediction accuracy of 57.1% for these layers. In contrast, the geological sweet spot standard identified no Class III layers in this context, highlighting the validity and effectiveness of the comprehensive fractal evaluation method.

### Machine learning integrated sweet spot prediction based on fractal characterization of reservoir physical parameters

The production capacity of fractured sections in shale reservoirs depends not only on the geological sweet spot evaluation but also on optimizing fracturing construction parameters. This machine learning approach adopts the random forest algorithm to build two prediction models for shale oil well fracturing production capacity^12^. It collects data on fracturing construction parameters from the CP2 and CP4 well segments, geological interpretation physical property parameters, and fractal parameters from extracted logging attributes. The models, prediction model I and prediction model II, are constructed using the attribute parameters listed in Table 5.

**Table 5:** Attribute parameter design of machine learning-based fracturing production capacity prediction model for shale oil wells.

Sports event	Geological parameter	Fracturing construction parameters
Predictive model I	Porosity, permeability, oil saturation, brittleness index	Total sand volume, cluster spacing, average sand ratio, total fluid volume, 70–140 mesh quartz sand, 40–70 mesh quartz sand, 30–50 mesh ceramic grains, fracturing section length
Predictive model II	Multiple fractal spectral widths (∆α1, ∆α2, ∆α3) for sonic, neutron, and density logging, the Fractal dimensions (D1, D2, D3) for sonic, neutron, and density logging	

**Fig. 6:**
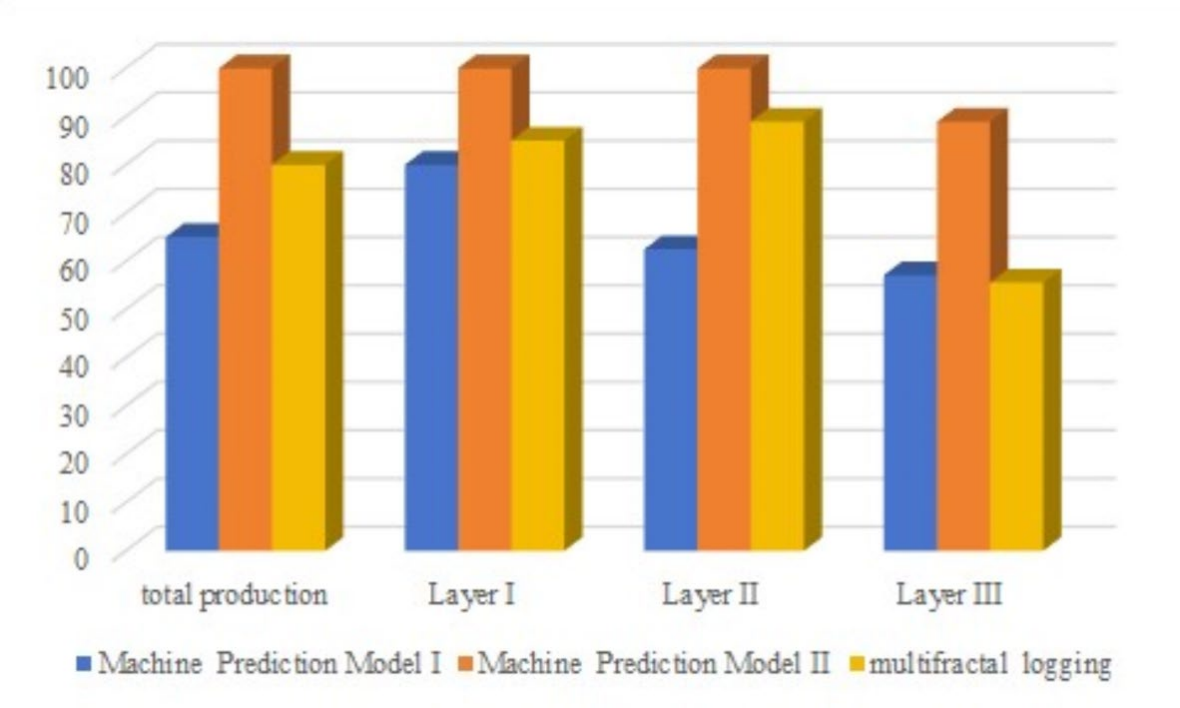
Accuracy comparison diagram of three productivity prediction methods.

The results of comparing the multiple prediction models are shown in Fig. 6. Overall, the prediction accuracy of Prediction Model II for the overall 22 sections of the H14–2 well in the Yangpai 2 platform is 95.5%, which is 18.2% higher than that of the 77.3% prediction accuracy of the multifractal spectral interpretation method, and 31.9% higher than that of the 63.6% accuracy of Prediction Model I.

For the prediction of the middle-class II layer (a total of nine segments), the Prediction Model II still leads with 100% accuracy, while the prediction accuracy of the multifractal spectrum interpretation method is 88.9%, and the accuracy of Prediction Model I is 62.5%. Although the accuracy of the latter two is slightly lower than that of Prediction model II, they still have high reliability in the prediction of the middle-class II layer.

In the prediction of the low-yield class III layer (a total of seven segments), the accuracy of Prediction model II is 88.9%, showing good prediction performance. The prediction accuracy of the multifractal spectrum interpretation method is 57.1%, while the accuracy of Prediction model I is 55.6%. This shows that although the prediction of low-yield layers is difficult, the prediction accuracy of Prediction model II is still high.

The reason for the low prediction accuracy of the models for the low-yield class III layers can be traced back to the tracer interpretation results for four sections (the 4th, 5th, 11th, and 15th sections, respectively). In these sections, prediction model II indicates low-yield oil contribution for the 4th, 5th, and 11th sections, and medium-yield oil contribution for the 15th section. In contrast, the multifractal spectrum interpretation method classifies these sections as Class III, Class II, Class II, and Class II, respectively. Prediction Model I consistently identifies these sections as Class II. These differences highlight the limitations and differences between prediction methods in assessing low-yield data.

Through in-depth analysis of these prediction results, we can not only understand the advantages and limitations of different prediction methods but also provide valuable reference and guidance for future research and practice. At the same time, the results also emphasize the necessity for further optimizing models and methods for predicting low-yield layers.

### Conclusion

1. Aiming at the problem of low accuracy of comprehensive sweet spot prediction in conventional geological evaluation, a comprehensive sweet spot evaluation method for fractured horizontal wells in shale reservoirs based on fractal theory to extract fractal characteristics of conventional logging curves is proposed. Taking the post-fracturing production profile as the constraint, the weighted multifractal spectrum width and weighted fractal dimension are introduced by using the grey correlation analysis method, and the three types of productivity evaluation criteria of fractured horizontal well section characterized by fractal are formed. The fractal evaluation method of comprehensive sweet spot section after fracturing of shale oil wells is established, which provides a new way for the accurate identification of sweet spots in shale reservoirs.
2. Based on the proposed fractal comprehensive evaluation method, it is verified in other fractured horizontal wells in the same sweet spot area, and the overall accuracy is 45.4% higher than that of the conventional geological evaluation method. By introducing the random forest algorithm combined with logging fractal characteristic parameters and fracturing construction parameters, a comprehensive sweet spot prediction model for fracturing is established, and the prediction accuracy is improved to 95.5%.
3. This method needs to use the post-fracturing production profile data to reflect the comprehensive sweet spot information, which is of great significance for identifying high-quality shale reservoirs and improving the development efficiency of shale reservoirs, and can provide strong technical support for the effective development of shale reservoirs.

## Data Availability

The datasets used and/or analysed during the current study available from the corresponding author on reasonable request.
